# Cholesterin Crystals in the Vitreous Humour of the Eye in a Horse

**Published:** 1884-01

**Authors:** 


### CHOLESTERIN CRYSTALS IN THE VITREOUS HUMOR OF THE EYE IN A HORSE.

---

After mentioning the same circumstance in the eye of a pigeon, Dr. Berlin says:

A horse was recently sent to me that had become blind in the right eye from frequent attacks of irido choroiditis. The other eye appeared healthy when observed without artificial assistance. It should be especially mentioned that no indications of anticipatory irido-choroiditis on this eye could be distinguished in any way. The pupil was of normal sise and without attachments in any way. Opthalmoscopic examination, however, revealed a most remarkable picture. The entire visible focus appeared to be filled with numerous small, bright refracting points of a pale yellow or white color, as if the fundus of the eye was completely covered with small, bright stars. They appeared to move with every movement of the eye; when the movement stopped they appeared to make a slight retrograde motion. These points appeared to have all about the same size and color. The normal fundus could be seen through this starry firmament.

This condition, which has not hitherto been described in the horse, is known in human opthalmology as "synchysis scintillans," or the accumulation of cholesterin crystals in the vitreous humor.

